# Comment on ‘Efficacy of electroacupuncture in improving postoperative ileus in patients receiving colorectal surgery: A systematic review and meta-analysis’

**DOI:** 10.1097/JS9.0000000000000972

**Published:** 2023-12-04

**Authors:** Xian-Wen Liang, Liang Chen, Jin-Cai Wu

**Affiliations:** Department of Hepatobiliary and Pancreatic Surgery, Hainan General Hospital (Hainan Affiliated Hospital of Hainan Medical University), Haikou, Hainan Province, People’s Republic of China

*Dear Editor,*

We have read with great interest the recent article by Chen *et al*.^1^ regarding the treatment of postoperative ileus using electroacupuncture in patients undergoing colorectal surgery. Based on a comprehensive analysis of 16 randomized controlled trials (RCTs) involving a total of 1562 patients, the authors discovered a significant association between the use of electroacupuncture and improved gastrointestinal functional recovery as well as reduced pain severity following colorectal surgery. We extend our sincere congratulations to the authors for publishing an outstanding systematic review and meta-analysis in the *International Journal of Surgery*. However, there are several aspects that warrant further discussion.

First, 2 months prior to the publication of this study, a similar meta-analysis^2^ was conducted to investigate the efficacy of acupuncture in treating postoperative ileus after colorectal cancer surgery. Among the studies included, one RCT^3^ met the inclusion criteria for this study; however, it was not incorporated into the current analysis, potentially impacting the reliability of the present findings.

Second, the articles included in the present pooled analysis were carefully examined, and it was identified that one study^4^ was not pertinent to the topic of this research. Therefore, further verification by the authors is required.

Third, based on the inclusion criteria outlined in this paper, it appears that all patients undergoing colorectal surgery fulfill the specified criteria for inclusion. However, it is imperative to enhance the study’s rigor as it specifically targets patients with colorectal cancer.

Finally, we recommend that authors employ the more objective Begger’s and Egger’s tests to identify potential publication bias in meta-analyses instead of relying on a visual assessment of funnel plot symmetry.

Overall, we express our heartfelt gratitude to Chen and colleagues for their concerted efforts in investigating the efficacy of electroacupuncture in improving postoperative ileus following colorectal surgery. They have taken a significant stride forward in this clinically relevant field. However, we would like to raise some concerns that could potentially assist the authors in enhancing the clarity of their findings.

## Ethical approval

No need for this Letter to the Editor.

## Sources of funding

None.

## Author contribution

X.-W.L. and L.C.: original draft conception and writing; J.-C.W.: critical revision of the manuscript. All authors reviewed the manuscript.

## Conflicts of interest disclosure

The authors have no conflicts of interest to declare.

## Research registration unique identifying number (UIN)

1. Name of the registry: none.
2. Unique identifying number or registration ID: none.
3. Hyperlink to your specific registration (must be publicly accessible and will be checked): none.

## Guarantor

Jin-Cai Wu.

## Data availability statement

None.

## Provenance and peer review

Commentary, internally reviewed.
